# FEASIBILITY AND ACCEPTABILITY OF A HYBRID AEROBIC EXERCISE PROGRAM FOR OLDER ADULTS DURING THE COVID-19 PANDEMIC

**DOI:** 10.1093/geroni/igac059.2889

**Published:** 2022-12-20

**Authors:** Emily Erlenbach, Revati Malani, Edward McAuley, Neha Gothe

**Affiliations:** University of Illinois, Urbana Champaign, Urbana Champaign, Illinois, United States; University of Illinois--Urbana-Champaign, Urbana Champaign, Illinois, United States; University of Illinois, Urbana Champaign, Urbana Champaign, Illinois, United States; University of Illinois at Urbana Champaign, Urbana, Illinois, United States

## Abstract

The COVID-19 pandemic and ensuing lockdowns, physical distancing and mask mandates exacerbated the challenges older adults (OAs) face towards exercise engagement. We present data on the feasibility, safety, and acceptability of a hybrid (in-person and virtual) aerobic exercise program for OAs. Nf39 (30 females, Mage=64.10) low-active OAs completed an aerobics-based exercise program as part of a larger ongoing RCT. Participants exercised 3x/week by attending one in-person and two Zoom-based exercise classes. Attendance, attrition, format preferences, and adverse events were documented. Participants also completed an anonymous survey to detail their experiences with the hybrid delivery model. Thirty participants completed the program. Total average attendance of 83.64%; 58.93% and 24.71% of the attended sessions were on Zoom and in-person, respectively. On post-program surveys, 36.67% reported preferring Zoom sessions, followed by 33.33% preferring both formats equally. No adverse events were reported. From the anonymous program feedback surveys, common reasons for preferring the Zoom sessions included convenience; not having to wear a mask; not worrying about COVID exposure; and not feeling self-conscious about exercising with others. Commonly reported reasons for preferring the in-person sessions were increased motivation from group energy; social support; more space to move around; and better engagement with instructors. Collectively, these findings show a hybrid aerobic exercise program is feasible and safe for OAs to engage in and is overall well-accepted. Findings are encouraging for the design of future exercise programs for OAs, who continue to remain a vulnerable population during the pandemic but need a structure to remain sufficiently active.

